# A normative framework for artificial intelligence as a sociotechnical system in healthcare

**DOI:** 10.1016/j.patter.2023.100864

**Published:** 2023-11-10

**Authors:** Melissa D. McCradden, Shalmali Joshi, James A. Anderson, Alex John London

**Affiliations:** 1 Department of Bioethics, The Hospital for Sick Children, Toronto, ON, Canada; 2 Genetics & Genome Biology Research Program, Peter Gilgan Center for Research & Learning, Toronto, ON, Canada; 3 Division of Clinical & Public Health, Dalla Lana School of Public Health, Toronto, ON, Canada; 4 Department of Biomedical Informatics, Department of Computer Science (Affliate), Data Science Institute, Columbia University, New York, NY, USA; 5 Institute for Health Policy, Management, and Evaluation, University of Toronto, Toronto, ON, Canada; 6 Department of Philosophy and Center for Ethics and Policy, Carnegie Mellon University, Pittsburgh, PA, USA

## Abstract

Artificial intelligence (AI) tools are of great interest to healthcare organizations for their potential to improve patient care, yet their translation into clinical settings remains inconsistent. One of the reasons for this gap is that good technical performance does not inevitably result in patient benefit. We advocate for a conceptual shift wherein AI tools are seen as components of an intervention ensemble. The intervention ensemble describes the constellation of practices that, together, bring about benefit to patients or health systems. Shifting from a narrow focus on the tool itself toward the intervention ensemble prioritizes a “sociotechnical” vision for translation of AI that values all components of use that support beneficial patient outcomes. The intervention ensemble approach can be used for regulation, institutional oversight, and for AI adopters to responsibly and ethically appraise, evaluate, and use AI tools.

## The bigger picture

How to holistically evaluate artificial intelligence (AI) tools in healthcare remains a challenge. Frameworks like regulatory guidelines and institutional approaches have focused narrowly on the tool’s performance alone, but we know that proper use of tools requires knowing the right way to use it and under what conditions. Tools are not neutral—they reflect our values. So, AI tools can be considered “sociotechnical systems”—meaning that their computational functioning reflects the people, processes, and environment. A “normative framework” for AI tools in healthcare supplies the practical guidance for operationalizing our values that incorporates not just the tool’s properties but the systems surrounding its use to achieve benefit.

## Teaser

Reliable, beneficial use of artificial intelligence (AI) in medicine requires the evaluation of its performance and the conditions that are linked with relevant outcomes. Rather than narrowly focusing on evaluating the system, the intervention ensemble framework motivates the evaluation of AI within a network of surrounding conditions that, together, produce benefit to patients and health systems. The authors explore this framework through the use case of an AI tool for detecting diabetic retinopathy.

## Introduction

It can be challenging to assess whether new artificial intelligence (AI) or machine learning (ML) healthcare applications promote the legitimate interests of patients and health systems. Stakeholders need a normative framework that can both assist them in navigating challenges and provide benchmarks against which new applications can be evaluated. Normative frameworks that provide guidance about algorithmic development tend to narrowly focus on attributes of the AI model, neglecting knowledge, practices, and procedures that are necessary to fruitfully integrate the model within the larger social systems of medical practice. The challenges and frictions associated with integrating these tools into clinical practice have been fruitfully studied by social scientists, who understand AI as part of a sociotechnical system.^1^ However, this work tends to be retrospective and descriptive. We propose a normative framework for advancing the responsible integration of AI systems into healthcare that captures the status of ML models as key pieces of a larger sociotechnical system. Using the concept of an intervention ensemble, we argue that AI systems should be evaluated as one element within a larger ensemble of knowledge, practices, and procedures that are jointly necessary to ensure that these innovations advance the legitimate interests of patients and health systems.

To motivate our perspective, we turn first to the current approaches to responsible translation. Guidance surrounding the development of ML models focuses heavily on the model as the main product of translation. Work in this area includes roadmaps and frameworks for responsible translation, the regulatory landscape, institutional governance, and explainability/interpretability.^2^^,^^3^^,^^4^^,^^5^^,^^6^^,^^7^^,^^8^ Typically, the goal in these frameworks is to identify the scientific practices necessary to make and maintain a “good” model. Although what makes a model good takes on a number of different meanings, this work is often limited to measures that narrowly focus on characteristics of the model and its outputs. These include criteria such as reliability, reproducibility, true/false negatives/positives, and a variety of technical measures of accuracy. Although clinical relevance is often emphasized as a criterion for evaluating model quality,^9^^,^^10^ this is often reduced to ensuring that the model is accurate at a prediction problem that clinicians feel is important.

Regulatory frameworks increasingly include rules requiring evidence for good clinical performance of models^11^—a welcome improvement given work demonstrating that a large proportion of FDA-approved AI systems have been granted on the basis of retrospective performance alone.^12^ However, regulatory bodies have not gone so far as to specify the methodology (e.g., prospective, controlled, quasi-interventional clinical trials) by which evidence is gathered. There is substantial variability regarding how performance should be assessed, which methods of evaluation to use, and what measures of performance are best.

Other work aimed at promoting responsible AI development focuses on producing models that are trustworthy, interpretable, or explainable, which we define broadly here as any attempt to support the users’ understanding of the way a model generates outputs from a set of inputs. Adjuncts like the “Model Facts” label offer information to satisfy the need for basic knowledge about the model and its validation process.^13^ Efforts in post hoc explainability are sometimes proposed as answers to questions about responsible clinical decision-making,^14^^,^^15^ but others have pointed out the computational^16^ and ethical^17^^,^^18^ limitations of current explainability methods for satisfying ethical decision-making in medicine. Others have remarked that explainability is not strictly needed to encourage clinical use of model outputs.^1^ Common among trustworthy, interpretable, and explainable approaches is the presumption that understanding how a model made a particular prediction is sufficient for responsible use.

In contrast, social science work has rejected a narrow view of AI as a technical product in favor of a broader frame in which these products are part of a larger sociotechnical system.^1^^,^^13^ For example, Madeleine Clare Elish’s unique work documenting the social dimensions of SepsisWatch demonstrated that its efficacy was heavily reliant on the work of the nursing staff charged with encouraging care teams to attend and respond to model outputs.^19^ Sandhu et al.^20^ observed that a model’s perceived clinical utility would be related to its overall ability to support the management of a clinical problem in a given unit rather than specific performance metrics. Henry et al.^21^ remarked that clinicians often did not feel the need to understand how a model arrived at its predictions to use it effectively, but they also noted that clinicians held inaccurate beliefs about the model’s capabilities. These descriptive findings demonstrate how clinicians can develop schemas to feel confident using ML tools at the point of care, predominantly drawing from their own personal observations of model performance and secondary mechanisms such as trustworthiness. However, they also highlight the lack of an evidence-based foundation for clinicians to draw from in using ML tools at the bedside. There is a limited understanding of the knowledge, practices, and procedures necessary for stakeholders to use AI systems to produce value for patients.^22^

To bridge this gap, we suggest a recognition of ML tools as one element within a larger intervention ensemble.^23^^,^^24^^,^^25^ This broader conception connects the fields of responsible AI and social sciences. The framework we propose is normative in the sense that it is offered as a guide to both facilitate responsible development of AI systems and to evaluate the decisions and practices of stakeholders who are developing, procuring, or implementing them. We hope that this framework is also relevant to shaping emerging commitments from regulatory bodies exploring the kind of evidence that should be collected to support the oversight of AI systems. Additionally, given that recent systematic reviews identified that two-fifths of AI tools evaluated through clinical trials fail to demonstrate superiority to standard of care,^26^ we hope that this framework offers a means to lend precision to prospective evaluation so as to increase the likelihood of positive results, thereby reducing research waste.^27^

## The intervention ensemble with healthcare ML

The intervention ensemble evolved from the recognition that interventions like drugs^23^^,^^24^^,^^25^ and other technologies such as autonomous vehicles^28^ are incapable of producing clinical benefit on their own. To realize their benefits, they must be embedded within an ensemble of knowledge, practices, and procedures that govern the use case for the intervention and include the conditions under which the intervention is likely to be effective, ineffective, or harmful and the steps that are required to monitor safety and efficacy.

Interventions, drugs, devices, and other tools cannot further the interests of patients unless they are used appropriately. For example, for pharmaceuticals, whether they have no effect, produce fatal toxicities, or confer clinical advantage is a function of a set of parameters including the indication(s) for which the drug provides benefit, the dosage at which the drug provides benefit, the window above which it is toxic and below which it is ineffective, the schedule on which the drug must be provided, and any additional diagnostic criteria needed to specify the populations most likely to benefit or be harmed by the drug. The intervention ensemble consists of the intervention itself (the drug) plus the set of parameters that modulate its effects in practice. Exploratory clinical research identifies (1) the boundaries within which the intervention is clinically useful and outside of which it is harmful and (2) approximate optimal values on key dimensions. Once these windows and optima have been identified, prospective confirmatory trials ascertain whether this intervention ensemble confers clinical benefit and under what conditions.^23^^,^^24^^,^^25^

Like pharmaceuticals, ML models alone do not improve outcomes for patients. Our contention is that responsible clinical development involves (1) identifying and properly characterizing the ensemble of knowledge and practices that must be understood and enacted if the system is to be used for clinical benefit and then (2) generating the evidence necessary to substantiate the claim that this ensemble is likely to produce a net benefit relative to alternative approaches in clinical practice.

To explore the idea of ML models as elements within an intervention ensemble, we conducted a narrative search of existing regulatory frameworks and reporting guidelines to identify components that each deemed important to translation of ML products. While there are many proposed frameworks to facilitate translation of ML,^29^ we chose to prioritize those which are predominantly oriented toward the explicit aim of evidence collection to support integration.^30^^,^^31^^,^^32^ We also drew from published reporting guidelines evaluating AI in clinical settings^33^^,^^34^^,^^35^ as these are explicitly geared toward establishing the required knowledge for clinical adoption. Further, drawing from some of these authors’ own experiences translating models at the point of care, we considered the set of elements that clinicians felt were necessary to support them in making decisions at the bedside.

**Figure 1:**
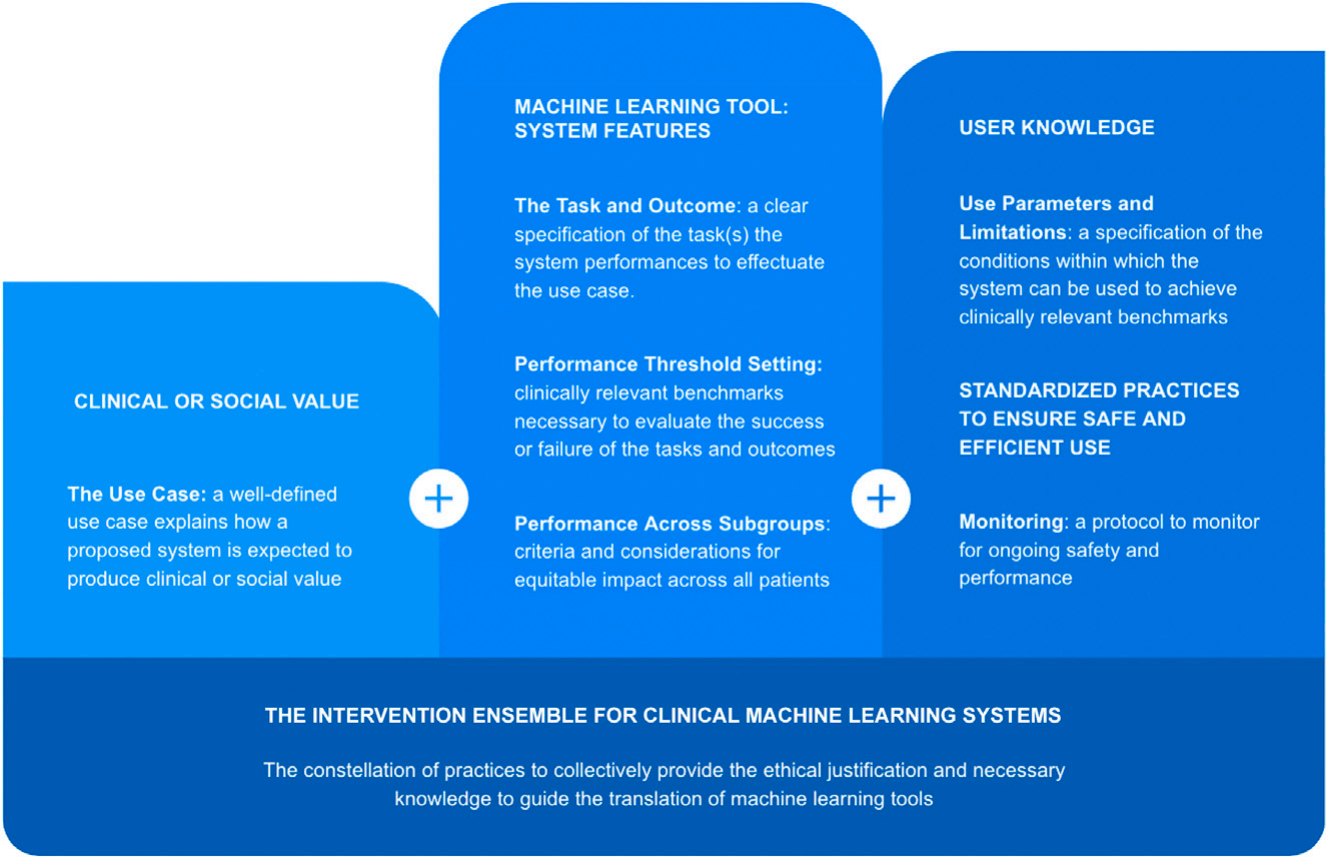
The intervention ensemble of clinical machine learning systems

We identified the following set of elements that would surround the use of an ML model and constitute the intervention ensemble for said model’s clinical use (Figure 1). Prioritizing the collection of this constellation of information as the product of a translation process for AI systems establishes the necessary and relevant set of information to guide clinical use.

(1) The use case: a well-defined use case explains how a proposed system is expected to produce clinical or social value by advancing patient interests or enhancing the capability of health systems to function more efficiently or more equitably.
(2) The task and outcome: a clear specification of the task(s) the system performs to effectuate the use case, including the way system outputs are to be integrated into clinical decision-making, practices, or procedures.
(3) Performance threshold setting: clinically relevant benchmarks necessary to evaluate the success or failure of the tasks and outcomes a system is designed to generate, their ability to achieve the intended use case, and the system’s ability to produce the desired clinical benefits relative to relevant alternatives.
(4) Performance across subpopulations: criteria and considerations for equitable performance and system use across the diversity of populations whose care may be influenced by the system’s outputs.
(5) Use parameters and limitations: a specification of the conditions within which the system can be used to achieve such benchmarks and outside of which its performance is expected to degrade, along with protocols or practices for implementing the system within these conditions under real-world conditions.
(6) Monitoring: a protocol for monitoring systems that are deployed in practice to ensure that their use satisfies these conditions and that changes in the clinical environment or updates to the system do not degrade its performance.

A case study approach of the IDx-DR system illustrates how an intervention ensemble might work. We chose IDx-DR because it is a widely recognized AI system that has been at the forefront of advancing health AI and has been used as a case example for many seeking to develop best practices around evaluation and oversight. Additionally, there are numerous publications to draw from surrounding the development of IDx-DR, which enriches our ability to test the applicability of the intervention ensemble. While detailed exploration of IDx-DR allows us to explore the intervention ensemble across all phases of development and implementation, we have also supplemented this case study with additional examples from the literature to enrich the discussion of the intervention ensemble. The selection of IDx-DR should not be taken as an endorsement specifically, nor are we suggesting it sets the standard.

IDx-DR is an FDA-approved AI system intended to screen for and detect mild diabetic retinopathy.^36^ The outputs are provided at the point of care accompanied by a recommendation for further evaluation by a specialist or a 6-month follow-up scan. IDx-DR’s approval followed a large observational trial testing the accuracy of its diagnostic properties.^36^ Table 1 depicts an intervention ensemble for IDx-DR.

**Table 1:** The intervention ensemble for IDx-DR: Linking the intended goals and benefits of the system with the evidence base to warrant empirical claims

	Rationale for thinking this system can be incorporated into practices and procedures that provide a specific benefit to patients/health systems	Evidence base to warrant relevant claims
Use case	detection of mtmDR in non-expert settings can facilitate timely referral for specialist-level care in the hopes of obtaining early treatment and minimizing diabetes-related vision complications	real-world impact on aggregate outcomes of health system efficiency (e.g., access to specialist care, speed of referral) has not yet been studied; no patient outcomes have yet been directly evaluated, such as access to DR-related care or a reduction in diabetes-related ophthalmological problems
Task and outcomes	automated detection of mtmDR among adults with diabetes not previously diagnosed with DR; system outputs are (1) positive for mtmDR, recommended referral to ophthalmology, or (2) negative for mtmDR, recommend re-screening in 12 months	prospective evaluation of the true clinical accuracy of the system’s ability to detect mtmDR across 10 primary care sites; the outcome assessed was the system’s performance against the reference standard, established by an expert panel; established the ability of non-specialist personnel to use IDx-DR in a clinical setting to detect mtmDR
Performance threshold setting	comparison to ophthalmologist accuracy rates at detecting mmDR under analogous conditions, as established by previous studies	evaluation demonstrated the accuracy of mmDR identification to be as follows: sensitivity 87%, specificity 90%, positive predictive value 73%, negative predictive value 96% (based on a prevalence of 24% for minimal DR)
Performance across subgroups	evaluate system performance across a range of relevant patient characteristics to detect differences in performance in anticipated subgroups	subgroup analysis for sensitivity and specificity by race, ethnicity, and sex to be equivalent; mild increase in specificity for adults >65 years of age; other metrics (e.g., failure case analysis) not reported
Use parameters and limitations	evaluate the relevant population and establish pertinent contraindications, warnings, standard operating procedure requirements, quality control measures, and conditions where the system fails	trial included patients with diabetes not previously diagnosed with DR, using standardized camera and equipment, using non-specialist technicians from study sites with 4 h of training, in a primary care setting; evaluated rate of images judged to be of insufficient quality and number of attempts to capture a sufficient image
Monitoring	practices necessary to monitor system performance, anticipate distribution shift, or assess performance after system updates	described as a “locked algorithm”; we were unable to find documentation in the literature regarding ongoing safety assessments or whether the system is updated over time with new data

IDx-DR, product name for the AI tool; mtmDR, more-than-mild diabetic retinopathy.

### A well-defined use case

Per the FDA, “IDx-DR is indicated for use by health care providers to automatically detect more than mild diabetic retinopathy (mtmDR) in adults diagnosed with diabetes who have not been previously diagnosed with diabetic retinopathy.” The use case thereby clearly defines a particular population (adults diagnosed with diabetes who have not been previously diagnosed with DR) and the task the system performs (detect pathologies of the eye that are established as valid indicators of mtmDR). Outside of these parameters (e.g., used to detect DR in patients without diabetes or to detect other eye pathologies), the use of IDx-DR would not be indicated based on the same evidence.

Ensuring a tight, logical link between the computational task and the use case is ideal. Passi and Barocas^37^ describe “problem formulation” wherein the knowledge about the label being predicted should be legitimately linked to the clinical problem to be addressed. Defining the use case can be relatively straightforward or it can be more complicated. Though there are “gold standards” for a plethora of disorders, there are few tests that are perfectly sensitive and specific.^38^ In other contexts, such as predicting short-term mortality risk or long-term benefit, there may not be an objective expert consensus on how to define the outcomes. The use case as defined sets out the precise scope of the ML tool’s application.

### A clear specification of the relationship between the use case and the desired clinical benefit

The task performed by an ML model must be integrated into a larger chain of actions and decisions in a way that plausibly generates benefit. Because the relationship between diagnosis in a primary care environment and a relevant patient outcome (e.g., disease progression, time to access specialist care) was not directly tested in the trial of IDx-DR, an observational trial allows us only to hypothesize about the potential benefit. Keane and Topol^39^ note that while observational studies are valuable, “such studies will not address the issue of clinical effectiveness—do patients directly benefit from the use of such AI systems?”

There are many examples of diagnostic aids, tools, and systems that demonstrate strong accuracy but have failed to yield benefits to patients. Advanced screening for various cancers through the use of computer-aided detection is emblematic of this gap: we can reliably identify abnormalities, but given how many are benign, identification itself might only result in increased anxiety, low-value testing, and waste of healthcare dollars rather than a benefit to patients.^40^^,^^41^^,^^42^ Accordingly, to ensure appropriate use of healthcare resources and to practice evidence-based adoption of novel technologies, many strongly advocate for testing AI’s systems through prospective, interventional trials prior to scaled adoption.^39^^,^^43^^,^^44^^,^^45^

Prospective interventional trials are increasingly pursued to test the association between the use of AI systems and a relevant clinical outcome. These can be patient-centered (e.g., mortality) or clinician/workflow-centered. As an example of the latter, BoneXpert is a system that automates bone age calculation superior to the current standard and significantly speeds up a radiologist’s workflow.^46^^,^^47^ On the patient-centered side, recent studies have sought to explore the relationship between the use of these tools and in-hospital mortality.^48^ Ensuring clear articulation of what evidence is gathered during a particular clinical study, and how this evidence contributes to knowledge of the reliable conditions of the system’s use, is central to responsible use of AI.

### Clinically relevant benchmarks necessary to evaluate the success or failure of the use case and its ability to produce the desired clinical benefit

IDx-DR’s outputs were defined to be consistent with established, consensus-based grading protocols.^36^ The confirmation of the outputs was then established against expert performance by having images interpreted by three expert readers masked to the AI output, where a majority voting paradigm established the final diagnosis. The pre-set thresholds to define the success or failure of IDx-DR’s diagnostic capabilities accounted for poor image quality and defined success as exceeding 75% for sensitivity and 77.5% specificity with an appropriate sample size. The authors contextualize this performance in light of reports that under similar parameters, board-certified ophthalmologists achieve sensitivity rates between 33% and 73%.^36^

Similarly, for BoneXpert, clinical evaluations first compare the accuracy of its bone age estimation against the performance of radiologists using the gold standard approach.^47^^,^^49^ Secondly, they assessed the amount of time radiologists spend performing the task manually compared with the use of the tool. Both time to task completion and accuracy of the task are the benchmarks by which the system is judged to succeed or fail.

### Criteria for equitable clinical performance across the diversity of populations on which the system is likely to be used

While it is important to ensure that accuracy is established against a reference standard or current practice, it is nonetheless important to question whether the status quo itself is effective for all patients. Health disparities are noted in diagnosis, prognosis, and access patterns within medicine. When AI systems replicate these patterns accurately, at scale, we risk further entrenching disparities.^50^^,^^51^ It is therefore essential to evaluate the success or failure of an ML tool within the entire population in which it will be used, both in aggregate and in relevant subgroups.^50^^,^^52^^,^^53^ Prospective clinical evaluation focused on patient outcomes can more reliably identify whether a model’s impact is equitable or not. For example, in some cases, algorithmic approaches might be preferable to an existing standard.^54^ However, confirmation on patient outcomes is needed to assess whether a “fair” algorithm translates to fair treatment.^53^

The trial report for IDx-DR identifies no significant effects in the performance of the system observed according to race, ethnicity, or sex.^36^ They note a mildly increased specificity among those over 65 years of age. The authors described the demographic range of participants in the study according to age, sex/gender, and ethnicity/race and stated that the biomarkers of DR are considered “racially invariant.” Notably, it must be acknowledged that these are somewhat imprecise terms that are proxies for factors that causally influence the outcomes of interest. For example, “sex” is often captured by either the gender or sex specified on a health card or insurance documentation or by the clinician’s impression. These are distinct from features that may well influence outcomes, such as hormones, experiences of sexism, anatomy, etc., though reducing performance to certain markers risks inappropriately essentializing differences as a function of patient identity.^55^ Considering what is directly measured with a given label forms a part of a holistic assessment of the overall fairness properties of a given system.^52^

Health equity scholars are re-asserting their long-standing advocacy for moving away from a neutral approach that fails to recognize differences between patients on the basis of demographic factors.^56^^,^^57^^,^^58^ The importance of disaggregating prospective (clinical) model performance according to patient groups is increasingly recognized in medicine.^50^^,^^59^ Clinical trial reporting guidelines include provisions for dis-aggregated reporting as a standard item.^33^ In one example, while BoneXpert performs well overall, a prospective study noted a higher error rate among girls living in India.^60^ As a non-demographic example, a hip fracture algorithm was noted to perform less accurately when the bone or joint in question had some abnormality.^61^ Such granular data collection can more precisely inform the intervention ensemble.

### A specification of the conditions within which the system can be used to achieve such benchmarks and outside of which its performance is expected to degrade

An important component of social value is that the ML model’s use is valuable at a specific point in the care pathway. For example, Oakden-Rayner et al. identified that a model intended to detect the presence of respiratory conditions performed considerably better on patients with a chest tube—a treatment for respiratory conditions.^62^ The implication is that a deployed model would be less accurate earlier on in the clinical pathway (where the identification of respiratory issues may be more likely to result in a net benefit) and more accurate once treatment has already commenced for the conditions for which the model is purportedly being used to identify.

For IDx-DR, the trial report contains very clear information about the conditions under which the system was evaluated.^36^ Adult patients with asymptomatic, diagnosed diabetes were evaluated in a primary care clinic by non-experts who received a standardized level of training to perform the screen. The conditions surrounding the image capture are also standardized, including the camera, patient positioning, etc. This sort of standardization and transparent reporting may foster better understanding of the generalizability of models and model approaches.^63^^,^^64^

### A protocol for monitoring systems that are deployed in practice to ensure that their use satisfies these conditions and that updates to the system improve and do not degrade its performance

Much like prospective postmarketing surveillance is needed in the context of pharmaceuticals to assess ongoing safety, effectiveness, and adverse event monitoring, AI systems require analogous postdeployment monitoring for safety.^65^^,^^66^ Algorithmovigilance is particularly important for AI due to its sensitivity to local contexts, susceptibility to data shifts, changes in patient-level patterns, and the effects due to changes in environmental factors (e.g., policy changes, seasonality, etc).^67^ Practice shift can occur where AI systems may be used in patient populations for whom they were not initially intended, causing adverse events.^65^ Notably, algorithms will differ in the extent to which they are susceptible to drifts. Since IDx-DR is a “locked” algorithm, for example, we are unaware of documented discussions of such protocols in this context.

Model performance can deteriorate for a variety of reasons. “Distribution shift” occurs when the distribution of features obtained during the training and testing of a model shift or change in such a way that the model no longer performs as expected. The cause of distribution shifts are myriad, including changes in the patient population; changes in data acquisition devices; software updates to data storage systems like electronic health records (EHRs); seasonality of diseases; clinician and patient incentives; practice recommendations; and adverse events like the COVID-19 pandemic.^68^ ML systems can also suffer from runaway feedback loops. When data collected based on model predictions are used to update ML models, model decisions can be significantly biased, as identified in the case of predictive policing, where the model repeatedly recommends policing in Black neighborhoods irrespective of actual crime rate.^69^ Less is understood about the implications of such feedback loops in the healthcare context.^70^ Due to the heterogeneity and shifts in patient populations, maintaining institutional governance will remain important.^8^ The intervention ensemble for a given system is not static and should be updated as relevant.

## Discussion

Treating ML tools as one component of a larger ensemble of knowledge, practices, and procedures that make up a useful medical intervention broadens the scope of evaluation for clinical translation. The approach helps researchers and clinicians guide the design and conduct of prospective evaluation to provide credible evidence of clinical utility.^27^ The clear pre-specification and analyses of the various parameters surrounding a model’s use can improve the trustworthiness of the research process while minimizing research waste and increasing the likelihood of a positive trial result.^27^ Additionally, the intervention ensemble (rather than the model alone) can constitute a more patient-centered unit for monitoring, education, and governance.^8^

A great deal of work has revolved around demonstrating accuracy as a necessary and sufficient condition for clinical use. Once accuracy is established, researchers turn to building the trust and acceptance of the user (via, for example, explainable interfaces). But accuracy alone does not establish clinical effectiveness, and the “likeability” of a system is not a morally significant metric of trustworthiness. The intervention ensemble concept bridges the gap between the operating characteristics of a model and the relevant information clinicians need to advance the interests of patients. Clinicians do not require tools to be perfect in order to use them; rather, they need to know when they work well, when they do not, and how their net clinical advantage compares to other clinical alternatives.

Notably, the intervention ensemble defended here does not require AI systems to be interpretable by design or explainable by another system. As argued previously,^18^ many interventions in medicine lack these properties in the sense that their clinical benefits have been demonstrated in well-designed trials but we do not know the precise mechanism by which they bring about that effect. We aim to provide standards for AI systems that are symmetrical with those applied to other medical interventions—stakeholders require the knowledge necessary to implement them in practice, and they require credible evidence that in doing so, they will advance patient interests relative to available alternatives. This is not to say we oppose interpretable systems—like others, we regard transparency and interpretability as desirable features of AI systems. However, we remain concerned that discourse surrounding AI sometimes overstates the value of interpretability and explainability, as though these conditions are necessary or sufficient for responsible deployment of AI systems. Our position is that, although these traits are desirable, they are neither necessary nor sufficient *in and of themselves* for deployment, at least insofar as these traits require knowledge of how algorithms work that goes beyond what has been described in the present framework.

Finally, we have developed this framework with the hope that it will assist in the responsible development and implementation of clinical AI. At this time, however, we have not yet evaluated its impact (e.g., can use of the intervention ensemble prevent over- and under-reliance on ML outputs, promote ML acceptance, or offer acceptable transparency to patients?). This is a task for future work. Future work might also explore its generalizability across contexts or its inclusivity to emerging variations of AI (e.g., generative models). We are interested in feedback from our colleagues on the utility of this framing.

### Conclusion

Scholars have noted the chasm between current practices for developing ML systems in medicine and the evidentiary needs of key stakeholders. Bridging this gap is necessary to ensure that research in this area generates the benefits necessary to improve patient outcomes, improve the delivery of health services, reduce unwarranted variation in practice, and ensure that practices are grounded in credible evidence of safety and efficacy.^71^ The intervention ensemble concept can improve the validation of AI systems by better meeting the information needs of key stakeholders, from clinician users to patients and health systems administrators. Understanding ML models as one component of a larger intervention ensemble encourages stakeholders to specify the use case, its relationship to a clinical benefit, the performance threshold setting, criteria for equitable performance across subpopulations, parameters and limitations, and a protocol for monitoring. These components together can provide the necessary foundation for beneficial use and holistic governance of ML systems.^8^
